# A point-of-care global thrombosis test measuring occlusion time and endogenous lysis time may indicate thrombotic status

**DOI:** 10.2144/fsoa-2019-0052

**Published:** 2019-07-02

**Authors:** Junichiro Yamamoto, Nobutaka Inoue, Kazunori Otsui, Hideo Ikarugi, Muneshige Shimizu, Shinji Yamamoto, Masahiro Murakami, Yoshinobu Ijiri, Kjell S Sakariassen

**Affiliations:** 1 Kobe Gakuin University, Kobe 651–2180, Japan; 2 Department of Cardiovascular Medicine, Kobe Rosai Hospital, Kobe 651–0053, Japan; 3 Department of General Internal Medicine, Kobe University Hospital, Kobe 650–0017, Japan; 4 School of Economics & Management, University of Hyogo, Kobe 651–2197, Japan; 5 Department of Fisheries, Faculty of Marine Science & Technology, Tokai University, Shizuoka 424–8610, Japan; 6 Faculty of Sport Sciences, Nihon Fukushi University, Aichi 470–3295, Japan; 7 Laboratory of Pharmaceutics, Faculty of Pharmacy, Osaka Ohtani University, Osaka 584–8540, Japan; 8 Department of Health & Nutrition, Faculty of Health & Nutrition, Osaka Shoin Women’s University, Osaka 577–8550, Japan; 9 Str. Campo e Zampe 12, I–13900, Biella, BI, Italy

**Keywords:** blood flow, endogenous fibrinolysis, native blood, platelet aggregation, shear, thrombosis, thrombotic biomarker, thrombotic indicator

## Can a single molecular biomarker predict overall thrombotic status?

Much research to identify clinically useful molecular biomarkers that reflect overall thrombotic status has been performed over the last few decades. One of the most probable molecular biomarkers appears to be D-dimer, but this lacks specificity, with poor predictive value in arterial thrombosis [1]. It is doubtful whether any single molecular biomarker could predict a multifactorial event such as an arterial thrombotic event. Another important problem is that *in vitro* anticoagulation of blood sample affects the results of the test used and renders it rather unphysiological [2–8].

However, some promising nonanticoagulated global hemostasis and thrombosis tests have been developed and have been used successfully in diagnosis and medical discovery and developments [9–14]. These tests have been well developed and proven to be highly important for both diagnostic and therapeutic purposes. Blood is drawn directly from an antecubital vein over a prothrombotic surface consisting of either human type III collagen fibrils or human tissue factor/phospholipids, which promotes thrombus formation by activation of both platelets and coagulation. Thrombus formation is performed at vessel wall shear rates varying from 100 to 32,000 s^−1^. The impact of vessel wall shear rates on the various mechanisms of thrombus formation and on anticoagulant and antiplatelet agents are very high. Platelet activation is increased by increasing arterial wall shear rate, whereas coagulation is increased by low venous wall shear rates. These physical shear rate effects have important effects on the activities of many antiplatelet and anticoagulant agents. Also, impacts of cigarette smoking and physical activities on thrombus formation have been studied with this global nonanticoagulated blood test [15,16].

It should also be mentioned that the use of these human nonanticoagulated blood tests is of importance for the antithrombotic and hematologic pharma industry, since animal studies may not always predict the human reaction. Furthermore, implication of these models in the pharma industry has reduced costs of the discovery and developmental activities and the need for animal experiments.

## Suggestions from animal experiments

We have assessed the optimal clinical test using helium neon laser-induced thrombosis *in vivo* tests and shear-induced platelet-rich thrombosis/thrombolysis (fibrinolysis) *in vitro* tests with nonanticoagulated (native) blood sample in experimental animals.

We screened antithrombotic activity of fresh juice prepared from 15 carrot varieties and further analyzed antithrombotic activity of the juice from the selected three varieties before and after heat treatment at 100°C for 10 min by the shear-induced platelet-rich thrombosis/fibrinolysis *in vitro* test (global thrombosis test [GTT]). Results for the first variety (SAKATA-0418) obtained by GTT *in vitro* test predicted that this variety has antithrombotic activity before heat treatment due to shortened lysis time (LT), but prothrombotic activity after heat treatment due to shortened occlusion time (OT). The prediction was proved by helium neon laser-induced thrombosis *in vivo* test. The result for the second variety (SAKATA-0420) before heat treatment as obtained by GTT could not predict pro- and antithrombotic activities because OT predicted prothrombotic activity, but LT predicted antithrombotic activity. This was demonstrated to be prothrombotic activity by helium neon laser-induced *in vivo* test, that is, platelet-rich thrombosis activity (OT) was predominant over thrombolytic activity (LT). Results after heat treatment predicted that overall activity may be antithrombotic because OT was not changed but LT was shortened. This was demonstrated by helium neon laser-induced thrombosis *in vivo* test. The result for the third variety (SAKATA-0421) before heat treatment predicted antithrombotic activity due to prolonged OT and shortened LT. This was demonstrated by helium neon laser-induced thrombosis *in vivo* test. Results after heat treatment predicted antithrombotic activity due to shortened LT regardless of unchanged OT. This was demonstrated by helium neon laser-induced thrombosis *in vivo* test [17].

Overall, it was shown that shear-induced platelet-rich thrombosis/fibrinolysis *in vitro* tests using native blood (nonanticoagulated blood) are well-matched to helium neon laser-induced platelet-rich thrombosis *in vivo* test [17]. In addition, the GTT using native blood and enabling simultaneous measurement of platelet-rich thrombotic and fibrinolytic activities demonstrated that thrombotic status is governed by both activities, indicating that simultaneous measurement of platelet-rich thrombosis and fibrinolysis measurement is useful for treatment of patients with thrombotic diseases. We have published articles on this topic in humans as well [6–8,18,19].

## Comparison of thrombotic molecular biomarkers using anticoagulated blood sample & shear-induced thrombosis/fibrinolysis test using nonanticoagulated blood (native blood) sample

We compared molecular biomarkers, fibrinogen, Fibrinopeptide A (FPA), D-dimer and agonist-induced platelet aggregation tests using anticoagulated blood and a shear-induced thrombosis/fibrinolysis test (Thrombotic Status Analyser [TSA] Montrose Diagnostics Ltd, UK; shear stress: 400 dynes/cm^2^) using native blood samples in non-insulin-dependent diabetic and stroke patients [20].

The thrombotic status of diabetic patients classified into subgroups according to the criteria of nephropathy stages and stroke patients with hemorrhage, lacunar infarction or other complications was compared by biomarkers using anticoagulated blood and a shear-induced thrombosis/fibrinolysis test using native blood.

Collagen-induced whole blood platelet aggregation test showed lowered platelet reactivity in all subgroups compared with control, suggesting antithrombotic status. ADP-induced platelet aggregation test showed various results; prothrombotic or antithrombotic status or no difference compared with control. FPA measurement gave no informative results. D-dimer measurement is a sensitive test, but this test was not specific, therefore practically may not be useful for treatment of patients with thrombotic disorders. In addition, it is unclear that increase in D-dimer is due to enhanced fibrin formation or enhanced fibrin degradation, or both. This may make it difficult to treat patients with thrombotic disorders, that is, it is unclear whether treatment should be targeted toward fibrin formation or fibrinolysis. In addition, information on platelet reactivity, which plays a central role in arterial thrombosis, is lacking in D-dimer measurement tests.

In contrast, shear-induced thrombosis/fibrinolysis tests using native blood sample can clarify whether prothrombotic or antithrombotic status is due to platelet-rich thrombotic activity or fibrinolytic activity. GTT is sensitive and can show prothrombotic status after physiological stress such as over work. In contrast, the widely used tests PT and APTT could not show significant prothrombotic status [19].

A shear-induced platelet-rich thrombosis test using native blood was superior in detecting prothrombotic status compared with the agonist-induced platelet aggregation tests and molecular biomarkers. Neither FPA nor D-dimer proved to be a reliable biomarker of prothrombotic status. Extensive clinical research has been performed with GTT showing the clinical effectiveness of this test in identifying patients at high risk.

## Conclusion

The point-of-care shear-induced thrombosis and fibrinolysis test using native blood samples (GTT) can measure platelet-rich thrombotic and endogenous fibrinolytic activities and thrombus stability under blood flow [21]. Unique features of the GTT include the testing of native blood and initiation of thrombus formation solely by pathologically relevant high shear stress. Formation of fibrin-stabilized thrombus and its lysis are detected – more information can be found in [7].

We propose that the thrombosis OT and endogenous LT measured simultaneously in the GTT are good clinical indicators to assess multifactorial events of thrombotic status. GTT is useful for not only treating patients with thrombotic disorders including cancer-associated thrombosis, but also in prevention of diseases and applications in other fields such as agriculture, nutrition, exercise and other aspects of healthcare.
